# Wide-field OCTA Quantified Peripheral Nonperfusion Areas Predict the Risk of Subclinical Neovascularization

**DOI:** 10.21203/rs.3.rs-6182918/v1

**Published:** 2025-05-05

**Authors:** Thomas Hwang, An-Lun Wu, Yukun Guo, Tristan Hormel, Christina Flaxel, Merina Thomas, Steven Bailey, Dong-Wouk Park, Yali Jia

**Affiliations:** Oregon Health & Science University; Casey Eye Institute; Oregon Health & Science University; Oregon Health & Science University; Oregon Health & Science University

## Abstract

**Purpose:**

To demonstrate the capabilities of single-shot widefield swept-source OCT angiography (SS-OCTA) in detecting subclinical retinal neovascularization (RNV), quantifying nonperfusion areas (NPAs), and exploring the relations between NPA and subclinical RNV in eyes graded as nonproliferative diabetic retinopathy (NPDR).

**Methods:**

Eyes clinically graded as moderate to severe NPDR underwent SS-OCTA imaging. Expert graders identified subclinical RNV, defined as vessels with flow signal above the internal limiting membrane on OCTA that are not visible on dilated fundus examination. This identification was based on a combination of *en face* OCT, *en face* OCTA, and cross-sectional OCTA overlaid on OCT. NPA index was calculated as a percentage of automatically quantified NPA over area in the posterior pole, the mid-periphery, and the total imaged area.

**Results:**

Totally 37 eyes including 21 had severe NPDR and 16 had moderate NPDR. Subclinical RNV was present in 14 eyes (37.8%). The eyes with RNV had significantly higher mid-peripheral and total NPA indices but not in the posterior region (mid-peripheral NPA: 31.97% ± 7.02% vs. 24.80% ± 6.60%, *p*=0.041; total NPA: 27.96% ± 6.36% vs. 21.61% ± 5.65%, *p*=0.046). The total NPA index showed the highest diagnostic accuracy for subclinical RNV detection (AUC: 0.761, with a sensitivity of 64.3% and a specificity of 87% at a cutoff value of 28.84%).

**Conclusion:**

Widefield SS-OCTA can detect subclinical RNV. The eyes with higher mid-peripheral NPA indices are more likely to have subclinical RNV, indicating that the NPA index may be a useful biomarker for identifying eyes at risk of RNV.

## Introduction

Diabetic retinopathy (DR) is the leading cause of preventable blindness among working-age adults globally, driven by chronic hyperglycemia leading to progressive microvascular damage in the retina.^1, 2^ As DR progresses, retinal nonperfusion and ischemia contribute to the development of retinal neovascularization (RNV), a hallmark of proliferative diabetic retinopathy (PDR), which is associated with sight-threatening complications such as vitreous hemorrhage and tractional retinal detachment. The extent of retinal nonperfusion area has been shown to increase with the severity of DR classification, indicating a pathological relationship between microvascular ischemia and disease progression, ultimately driving DR toward more advanced stages.^3, 4^

Currently, the standard of care for detecting RNV is careful dilated ophthalmoscopy or color fundus photography according to American Academy of Ophthalmology Preferred Practice Patterns on diabetic retinopathy.^5^ Fluorescein angiography (FA) is usually used to identify suspected but clinically obscure RNV and is not routinely as a part of the regular examination of patients with diabetes. As a result, we may overlook subtle preclinical microvascular changes that are not clinically suspected. Optical coherence tomography angiography (OCTA)^6, 7^ offers advantage of effectively revealing vascular abnormalities including neovascularization on the retinal surface by providing non-invasive, high-resolution, three-dimensional visualization of retinal blood flow in relation to retinal structure, enabling the identification of subtle RNV in DR cases that were undetectable via clinical examination or color fundus photography.^8, 9^ This is a safer, faster, and less expensive alternative to FA for routine RNV screening, particularly in more advanced cases of nonproliferative diabetic retinopathy (NPDR), where the cost and risks associated with FA may outweigh the potential benefit of identifying unsuspected RNV. Our group demonstrated that combining *en face* OCT, *en face* OCTA, and cross-sectional OCTA significantly improves detection accuracy, allowing for the detection of occult RNV with high sensitivity.^10^ Additionally, we characterized distinct morphologies of RNV, identifying sprouts and fronds, with longitudinal studies highlighting their evolution and potential as biomarkers of disease progression.^11^ In this study, we define subclinical RNV as the identification of vessels with flow signal above the internal limiting membrane (ILM) on OCTA, which are undetectable through standard clinical examinations.

Despite these advances, most studies utilizing OCTA have been constrained by small fields of view that can overlook mid-peripheral lesions where early RNV often originates.^12^ The advent of single-shot widefield OCTA offers a transformative opportunity to investigate retinal changes more comprehensively.^13^ Moreover, a robust deep-learning-based quantification of nonperfusion areas (NPAs), which correlate with DR severity and are not affected by imaging artifacts, has been validated.^14, 15^ These innovations provide the opportunity to integrate the detection or RNV and quantification of NPA using OCTA widefield imaging to improve the detection and understanding of early neovascular changes in DR. This study explores the relationship between NPA and the presence of RNV using a single-shot widefield OCTA.

## Method

### Study Participants

We recruited patients with moderate to severe NPDR based on fundus examination using the Early Treatment of Diabetic Retinopathy Study (ETDRS) criteria for a prospective observational study (NIH grant R01 EY035410) at the Casey Eye Institute, Oregon Health & Science University. The study adhered to the tenets of the Declaration of Helsinki and complied with the Health Insurance Portability and Accountability Act of 1996. Approval was obtained from the Institutional Review Board of Oregon Health & Science University, and written informed consent was obtained from all participants.

Eligible eyes underwent widefield swept-source OCT angiography (SS-OCTA) imaging between December 2023 and September 2024. Eyes with significant concomitant ocular diseases or poor image quality due to motion artifacts or inadequate signal strength were excluded. If both eyes of a patient met the eligibility criteria, one eye was randomly selected for inclusion. All participants underwent comprehensive ophthalmic examinations, including standard ETDRS visual acuity testing, intraocular pressure measurement, slit-lamp biomicroscopy, indirect binocular ophthalmoscopy.

### Image Acquisition and Analysis

Imaging was performed using a pre-market commercial 200-kHz swept-source OCTA system (DREAM OCT, Intalight Inc.) with 1050 nm central wavelength. Each eye was centered on the fovea during imaging, and scans were acquired using the OCTA volumetric scans of 26 × 21 mm with 1536 × 1240 sampling density. These settings provided a field of view up to an eye angle of 130 degrees, enabling high-resolution imaging of both the posterior pole and mid-peripheral retina.

We used different segmentation strategies for *en face* OCT vs. OCTA to optimize the identification of RNV as previously reported in our earlier studies.^10, 11^ For *en face* OCT, the vitreoretinal interface slab was defined as the region bounded by the ILM and a position 30 μm anterior to the ILM. For *en face* OCTA, the slab was defined as the region bounded by the ILM and a position 300 μm anterior to the ILM. RNV was identified by the presence of flow signals above the ILM on cross-sectional OCTA (overlaid on OCT), corresponding to epiretinal hyperreflective material on *en face* OCT or abnormal flow signals on *en face* OCTA. The presence of RNV was determined by two trained graders (A.W. and Y.G.) and confirmed by an expert grader (T.S.H.) using a combination of *en face* OCT, *en face* OCTA, and cross-sectional OCTA generated with commercially available software.

To perform image processing for NPA quantification, the entire retinal layer was segmented from the ILM to the outer plexiform layer using a guided bidirectional graph search algorithm segmented the retinal layer boundaries.^16^ A maximum projection method was used to project OCTA data within the slab to visualize *en face* angiogram.^17^ An NPA map was created using a deep learning-based algorithm to automatically quantify NPAs as in previously reported methods.^14, 18–20^ The total NPA index defined as the percentage of quantified NPA over the total imaged area. We also analyzed the NPA index separately for the posterior pole, defined as the region centered on the fovea, with the radius defined by the distance from the fovea to the optic disc center, and the mid-periphery, defined as the area outside this circle. The foveal avascular zone was excluded from these calculations. The location of each RNV lesion was documented as either within the posterior pole or the mid-periphery of the retina, based on the same criteria, enabling further spatial analysis of lesion distribution.

### Statistical Analysis

Data were analyzed using SPSS Statistics (version 26.0; IBM Corp, Armonk, NY). Descriptive statistics are reported as mean ± standard deviation, and counts with percentages, were used to summarize demographic and baseline clinical characteristics as appropriate. Fisher’s exact test or Chi-squared test were applied to compare categorical variables, while independent t-tests were used for continuous variables. An area under the receiver operating characteristic curve (AROC) was analyzed to evaluate the diagnostic performance of NPA indices in classifying eyes with or without subclinical RNV. Sensitivity, specificity, and area under the curve values were calculated for posterior pole, mid-peripheral, and total NPA indices. The optimal threshold for NPA index associated with subclinical RNV detection was determined using the Youden Index. This threshold was then used to identify eyes at high risk for subclinical RNV development, with regional comparisons performed to determine the most diagnostically valuable metric. All *P* values were two-tailed, and a value of < 0.05 was considered statistically significant.

## Results

Of 38 initially enrolled eyes, 37 (97.4%) had capillary details with sufficient image quality for analysis and one was excluded due to significant motion artifacts and signal loss. Of these, 21 eyes (56.8%) were clinically graded as severe NPDR, and 16 eyes (43.2%) as moderate NPDR. The average age of participants was 56.9 ± 10.8 years, with a gender distribution of 14 males and 23 females. Among the study participants, 27 (73%) were treatment-naïve, defined as having no prior panretinal photocoagulation or anti-VEGF injections. The participant characteristics are summarized in Table 1.

Subclinical RNV was identified in 14 eyes (37.8%) using widefield SS-OCTA. Among these eyes, 6 cases (42.9%) exhibited RNV exclusively in the mid-periphery. Of the 14 eyes with subclinical RNV, 8 eyes (57.1%) were clinically graded as severe NPDR, while 6 eyes (42.9%) were graded as moderate NPDR. A comparison of the clinical characteristics between eyes with and without subclinical RNV revealed no significant differences (Table 2).

**Figure 1:**
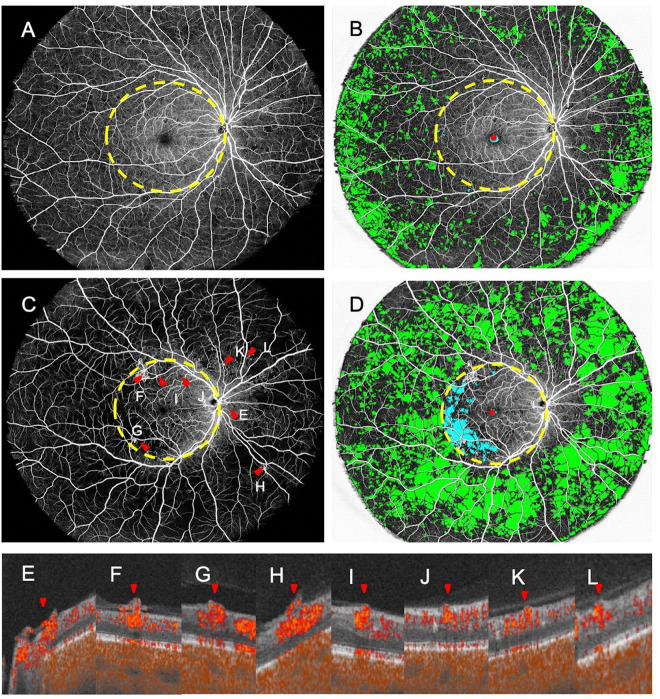
Representative *en face* angiogram of the entire retinal layer from cases with and without subclinical retinal neovascularization (RNV). Images are divided into posterior pole and mid-peripheral regions, delineated by a yellow dashed circle centered on the fovea, with the radius determined by the distance from the fovea to the optic disc center. Nonperfusion area (NPA) in the posterior pole and mid-peripheral retina is coded in blue and green, and the foveal avascular zone in red, which is excluded from the NPA calculation. (A, B) *En face* angiogram and NPA map of a 50-year-old male with moderate NPDR and no subclinical RNV. (C, D) *En face* angiogram and NPA map of a 54-year-old male with severe NPDR showing RNVs (red arrows). (E–L) RNV is visualized in OCT B-scans with flow overlaid showing abnormal flow signals breaching the internal limiting membrane.

The mean NPA indices (± SD) of the eyes with subclinical RNV for the posterior pole, mid-peripheral retina, and total retinal regions were 3.49 ± 3.25%, 31.97 ± 7.02%, and 27.96 ± 6.36%, respectively. The corresponding NPA indices for eyes without RNV were 1.74 ± 2.11%, 24.80 ± 6.60%, and 21.61 ± 5.65%. The total and mid-peripheral NPA indices were significantly higher for the eyes with RNV compared to those without (p < 0.05) but not the posterior NPA index. Representative images of eyes with and without subclinical RNV are shown in Fig. 1, along with corresponding nonperfusion maps featuring color-coded delineations of different retinal regions.

When comparing eyes with subclinical neovascularization at disc (NVD) vs. subclinical neovascularization elsewhere (NVE) only eyes, subclinical NVD eyes had significantly higher mid-peripheral and total NPA indices of 35.82 ± 5.86%, and 31.46 ± 4.93% vs. 29.48 ± 5.50%, and 25.67 ± 5.45% (p < 0.05). The posterior NPA indices for the two groups were not significantly different at 4.77 ± 3.50% for NVD eyes and 2.49 ± 2.75% NVE only eyes. The comparison of NPA indices among the groups is presented in Table 3.

**Figure 2:**
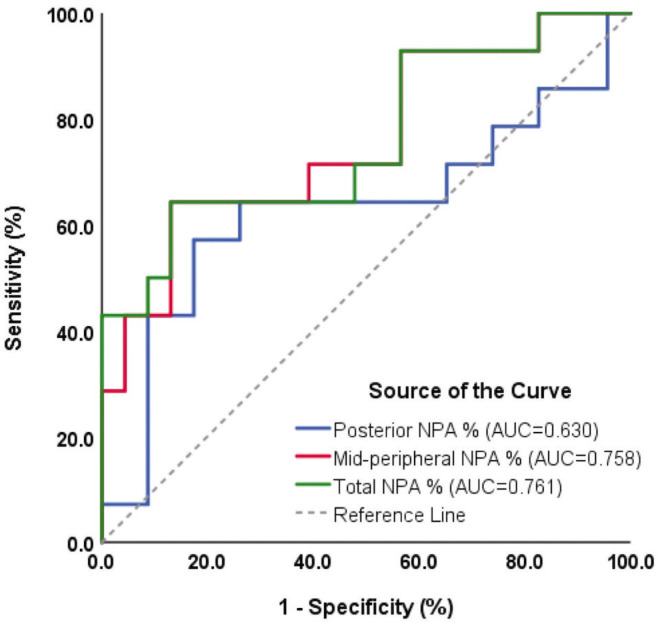
Receiver operating characteristic curves for evaluating the predictive accuracy of nonperfusion area (NPA) index in detecting subclinical retinal neovascularization (RNV). The green line represents the total NPA percentage (AUC = 0.761), the red line represents the mid-peripheral NPA percentage (AUC = 0.758), and the blue line represents the posterior NPA percentage (AUC = 0.630).

The total NPA index had the highest AUC for identifying eyes with RNV, with an AUC of 0.761 (95% CI, 0.592–929), compared to 0.758 (95% CI, 0.591–924) for the mid-peripheral NPA index and 0.630 (95% CI, 0.424–837) for the posterior pole NPA index (Fig. 2). Based on the Youden index, the optimal cutoff value for the total NPA index was 28.8%, achieving a specificity of 87% and a sensitivity of 64.3% for detecting subclinical RNV.

## Discussion

This study demonstrated the utility of single-shot widefield OCTA in detecting subclinical RNV in a significant number of eyes clinically graded as moderate or severe NPDR. In nearly half of these eyes, the subclinical RNV was found exclusively in the mid-periphery. Additionally, we demonstrated the value of peripheral NPA quantification using deep-learning algorithms, which predicted the presence of subclinical RNV better than the NPA assessment of the posterior pole. These findings highlight the diagnostic benefit of widefield OCTA in detecting clinically undetectable RNV, offering a clear advantage and potentially leading to significant improvements in diabetic retinopathy management.

Our study demonstrated a higher detection rate of subclinical RNV compared to previously published rates of 15%–28% reported in our prior work.^9–11^ While some of the differences may be explained because the current study included only the eyes graded as moderate to severe NPDR, the increased detection rate in this series may also be attributed to the use of a larger field of view. Although prior studies have utilized montage views of OCTA scans to achieve a widefield view (approximately 17 × 17 mm),^9–11^ our single-shot widefield imaging in this study captures a broader retinal area (26 × 21 mm), extending anatomically closer to the posterior edge of the vortex vein ampulla.^21^ This approach has the potential to provide more diagnostic information in a non-invasive and efficient manner. This reinforces the desirability of widefield OCTA as a tool for detecting proliferative changes in its earlier stages and exploring these preclinical abnormalities could facilitate better characterization of disease severity and identify new therapeutic targets.

The total and mid-peripheral NPA indices were significantly higher in eyes with subclinical RNV compared to those without, highlighting the critical role of retinal nonperfusion in DR pathophysiology and its potential as a biomarker for disease severity. FA-identified nonperfusion has been recognized as a hallmark of retinal ischemia, correlating with microvascular damage and an increased likelihood of neovascularization.^22, 23^ However, FA-based assessments are invasive and can pose challenges in accurately quantifying ischemia, especially when microvascular leakage is present. OCTA enables the quantitative detection of non-perfused areas without the confounding effects of leakage.^24^ Studies have validated that OCTA demonstrates superior sensitivity in detecting NPAs compared to FA and provides repeatable and reproducible automated metrics for evaluating retinal ischemia.^8, 25^ In this study, we calculated NPAs using the inner retinal slab because the superficial vascular complex and deep capillary plexus progressively merge as they extend beyond the posterior pole.^26^ This anatomical configuration makes capillary dropout more reliably visualized using *en face* OCTA of the entire inner retinal slab.^14^ Our previous research has demonstrated that NPAs detected using OCTA are significantly associated with DR severity, progression, and treatment requirements.^27^ These findings indirectly support our results, highlighting the potential of single-shot widefield OCTA as a non-invasive and accessible tool capable of detecting pathologic features that may not be readily visible to clinicians.

Retinal NPA location also appears to be relevant. In our study, nearly half of the eyes with RNV had lesions exclusively in the mid-periphery and the mid-peripheral nonperfusion emerged as a key factor for the development of neovascularization, consistent with previous studies suggesting that the mid-peripheral and far-peripheral retinal zones may be particularly sensitive to the impact of diabetic retinopathy.^4, 28, 29^ However, the literature is inconsistent in defining NPA localization, with some studies using radius-based definitions, while others adopt field-of-view concepts or the concentric ring method.^23, 29, 30^ In this study, we adopted an anatomically guided approach to define retinal regions, using the fovea as the center and the distance to the optic disc as a reference for segmentation. This approach better captures the physiological relevance of topographic differences, defining the posterior pole as the area just beyond the disc and vascular arcades, and the mid-periphery as the region extending to the posterior edge of the vortex vein ampulla, consistent with the eye angle 130-degree field of view provided by our widefield OCTA.^21, 31^

In eyes with subclinical RNV, we observed significant differences in total and mid-peripheral NPA indices between eyes with NVD vs. those with NVE only. Previous FA-based and OCTA-based studies reported comparable findings.^22, 32^ These findings may suggest that the presence of NVD is indicative of more severe ischemia burden. This may also explain the long-established observation that eyes with NVD are at a greater risk for severe vision loss compared to eyes with NVE only.^33^ Such cases may warrant closer monitoring and more aggressive treatment compared to eyes with NVE alone.

Subclinical RNV can be understood as subclinical precursors to clinically evident RNV.^11^ While clinical grading systems like the ETDRS can estimate the short and long term risk of proliferative disease, they are imprecise in that conversion to proliferative does not go always go through stepwise progression mild to moderate to severe NPDR and even mild NPDR can progress to PDR in one year. We theorize that eyes with subclinical RNV are at a higher risk of developing clinically evident RNV for the same level of clinical retinopathy severity level. By combining the detection of subclinical RNV and quantification of NPA, we may be able to more precisely predict which eyes are at risk for developing vision-threatening retinopathy compared to clinical grading. For instance, eyes approaching or exceeding the 28.84% threshold could be presumed at higher risk for developing overt RNV compared to eyes with a much lower NPA index, even if both are clinically classified within the same DR severity stage. Such thresholds could indirectly influence treatment strategies by identifying cases that may benefit from closer monitoring or earlier intervention. While these findings highlight the potential for subclinical RNV and NPA as a predictive biomarker, further larger and longitudinal studies are required to validate its prognostic utility and assess its role in guiding earlier interventions in diabetic retinopathy.

This study has several limitations that should be considered. First, image distortion remains a confounding factor due to the curved retinal surface and the challenges of imaging the peripheral retina. This distortion may have affected measurements of peripheral capillary nonperfusion, particularly in eyes with longer axial lengths, posing challenges for accurate intercapillary distance measurements and NPA calculations.^34^ To address this, we normalized NPAs using an index and defined retinal regions based on anatomical landmarks, such as the fovea and optic disc, ensuring anatomic region segmentation. However, future efforts to recognize and correct image distortion are necessary to improve measurement accuracy.^35^ The resolution of the single-shot 26 × 21 mm OCTA scans is lower than that scans of smaller fields of view, which may limit sensitivity to subtle microvascular abnormalities. Nonetheless, the broader field of view enabled a comprehensive assessment of ischemic changes across the retina. Additionally, not all cases were treatment-naïve, and some subclinical RNV may represent regressed neovascularization following prior interventions. This highlights the need for refined DR grading systems that account for documenting treatment responses, enabling a more accurate representation of disease severity and current activity.^36^ Finally, longitudinal studies with larger cohorts and advancements in imaging technology are essential to validate the predictive value of NPA indices and further enhance the clinical applicability of widefield OCTA in diabetic retinopathy.

In conclusion, this study demonstrates the utility of a widefield OCTA in detecting subclinical RNV and quantifying retinal nonperfusion areas in NPDR. By employing a single-shot imaging protocol and an anatomically guided approach to retinal region segmentation, we provided a comprehensive assessment of ischemic changes across the posterior and mid-peripheral retina. The identified NPA thresholds, particularly in the mid-peripheral region, highlight the potential of OCTA-derived metrics as predictive biomarkers for early proliferative changes.

## Figures and Tables

**Table 1:** Demographic and clinical characteristics of the study participants

Parameters	(n = 37)
Age, year	56.9 ± 10.8
Sex, n (male/female)	14/23
Laterality, n (right/left)	12/25
Most recent HbA1c, %	8.0 ± 2.2
Duration of DM, years	19.4 ± 8.0
Type 1 DM, n (%)	6 (16.2)
DR stage	
Moderate NPDR, eyes (%)	16 (43.2)
Severe NPDR, eyes (%)	21 (56.8)
Treatment naïve, eyes (%)	27 (73.0)
Previous treatment	
Intravitreal VEGF injection, eyes	8
Panretinal photocoagulation, eyes	0
Both, eyes	2
logMAR Visual Acuity	0.08 ± 0.18

HbA1c: Hemoglobin A1c; DM: diabetes mellitus; DR: diabetic retinopathy; NPDR: non-proliferative diabetic retinopathy; VEGF: vascular endothelial growth factor; logMAR: logarithm of the minimum angle of resolution.

**Table 2:** Characteristics between eyes with and without subclinical retinal neovascularization

Parameters	RNV(+) (n = 14)	RNV(−) (n = 23)	*P*-value
Age, year	56.9 ± 11.2	56.9 ± 10.8	0.929
Sex			0.804^†^
Female	8 (57.1)	15 (65.2)	
Male	6 (42.9)	8 (34.8)	
Laterality			0.367^†^
Right	10 (71.4)	15 (65.2)	
Left	4 (28.6)	8 (34.8)	
Most recent HbA1c, %	8.9 ± 2.9	7.5 ± 1.5	0.684
Duration of DM, year	20.4 ± 8.7	18.8 ± 7.8	0.568
DM Type			0.561^†^
Type I	1 (7.1)	5 (21.7)	
Type II	13 (92.9)	18 (78.3)	
DR stage			1.000^†^
Moderate NPDR	6 (42.9)	10 (43.5)	
Severe NPDR	8 (57.1)	13 (56.5)	
Treatment naïve			1.000^†^
No	2 (14.3)	8 (34.8)	
Yes	12 (85.7)	15 (65.2)	
logMAR Visual Acuity	0.14 ± 0.25	0.04 ± 0.11	0.445

RNV: retinal neovascularization; HbA1c: Hemoglobin A1c; DM: diabetes mellitus; DR: diabetic retinopathy; NPDR: non-proliferative diabetic retinopathy; logMAR: logarithm of the minimum angle of resolution.

† Fisher’s exact test

**Table 3:** Comparison of retinal nonperfusion areas between eyes with and without subclinical RNV, and betweeneyes with subclinical NVD and subclinical NVE

Parameter	RNV(+) (n = 14)	RNV(−) (n = 23)	*P*-value	NVD (n = 7)	NVE (n = 7)
Posterior NPA, %	3.49 ± 3.25	1.74 ± 2.11	0.328	4.77 ± 3.50	2.49 ± 2.75
mid-peripheral NPA, %	31.97 ± 7.02	24.80 ± 6.60	0.041	35.82 ± 5.86	29.48 ± 5.50
Total NPA, %	27.96 ± 6.36	21.61 ± 5.65	0.046	31.46 ± 4.93	25.67 ± 5.45

NPA: nonperfusion area; NVD: neovascularization of the optic disc; NVE: neovascularization elsewhere; RNV: retinal neovascularization.
